# Systemic Everolimus Does Not Block the Incubation of Methamphetamine‐Craving: A Dose–Response Study

**DOI:** 10.1111/adb.70189

**Published:** 2026-09-13

**Authors:** Fernando J. Cano, Kierra E. Smith, Katherine Stuemke, Asha Jotwani, Erin Lassers, Holly M. Nelson, Sonya D. Adler, Laura L. Huerta Sanchez, Christopher J. E. Denning, Samantha M. Davantes, Citlali A. Ibarra, Tod E. Kippin, Karen K. Szumlinski

**Affiliations:** ^1^ Department of Psychological and Brain Sciences University of California Santa Barbara Santa Barbara California USA

## Abstract

In rodent models, incubated craving for drugs and natural reinforcers is characterized by a time‐dependent increase in cue‐elicited operant responding, which posits that common biochemical mechanisms gate incubated craving across different types of reinforcers. Recently, our laboratory identified heightened mTOR activity in the prelimbic (PL) and infralimbic (IL) subregions of the medial prefrontal cortex (mPFC) as being associated with incubated craving for cocaine and sucrose, respectively. Importantly, Everolimus, an FDA‐approved mTOR inhibitor, blocked the expression of incubated craving for both reinforcers. Hence, our current study investigated whether mTOR signalling also underpins cue‐induced methamphetamine (METH)‐seeking by evaluating the “therapeutic potential” of Everolimus to block incubated METH‐craving. Male and female rats were trained under extended‐access procedures (6‐h/day × 10 days) to self‐administer intravenous METH (0.1 mg/kg/0.1 mL infusion), paired with the presentation of a 20‐s light + tone compound stimulus and then were subjected to a withdrawal period of 1 or 30 days (WD1 or WD30). Rats tested on WD30 were gavage‐infused with either vehicle (VEH; 1% DMSO in water; vol: 1 mL/kg) or Everolimus (1 or 10 mg/kg), whereas WD1 rats were infused with VEH to index baseline craving. Thirty minutes later, rats underwent a 2‐h test to measure cue‐elicited responding. Akt/mTOR activity in the PL and IL was examined via immunoblotting procedures. Male and female rats exhibited comparable METH intake and craving, with no effect of estrous cycle observed for either measure in females. Unlike previous findings for incubated cocaine‐ and sucrose‐craving, Everolimus did not reduce incubated METH‐craving. Further, incubated METH‐craving was not associated with changes in Akt/mTOR activation within mPFC subregions nor did Everolimus lower mTOR pathway activation below baseline levels in either METH‐experienced or –naive rats. Together, these findings demonstrate that elevated mTOR signalling within mPFC is not involved in incubated METH‐craving, highlighting the potential need for drug‐specific targeted treatments for curbing incubated craving in protracted withdrawal.

## Graphical abstract

Oral pretreatment with the mTOR inhibitor Everolimus failed to block incubated methamphetamine‐seeking in a rat model, even at doses as high as 10 mg/kg. Further, neither methamphetamine experience nor Everolimus pretreatment altered protein indices of mTOR signaling with the prefrontal cortex. Thus, unlike prior results for incubated cocaine‐ and sucrose‐craving, incubated methamphetamine‐craving does not involve mTOR activation within this addiction‐relevant brain region.

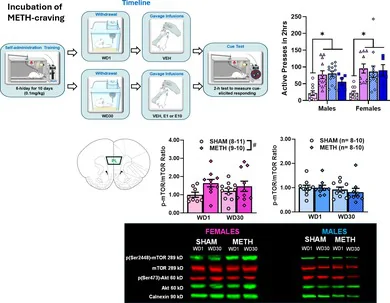

## 1 Introduction

The psychomotor stimulant methamphetamine (METH) is one of the most widely abused illicit drugs in the world and its chronic use is associated with profound psychological and physiological consequences, including neurotoxicity, psychiatric comorbidities, cognitive deficits, impaired decision‐making and high relapse rates [1, 2, 3]. One major contributor to the high relapse rates in METH use disorder (MUD) is craving [4, 5], which can be elicited by re‐exposure to METH‐associated cues [6]. Cue‐elicited craving is acute and intense [7] and can intensify over the course of the first several months into abstinence in those with MUD [8, 9]. Termed the incubation of craving [10], an analogous phenomenon is observed under various rodent models of intravenous (IV) METH self‐administration (e.g., [11, 12, 13, 14, 15, 16, 17]) and, as reported in humans [8, 9], can persist for 100 + days in rats [18].

It has been suggested that signalling pathways that promote protein translation, such as the mammalian target of rapamycin (mTOR) pathway, are required to maintain neuroadaptations that enable the expression of incubated craving [19]. In support of this, incubated cocaine‐craving by rats is associated with elevated indices of mTOR‐related signalling within both the nucleus accumbens [19] and the medial prefrontal cortex (mPFC) [20, 21, 22], with additional evidence linking incubated heroin‐craving to heightened activation of the upstream activator of mTOR, Akt, within the nucleus accumbens [23]. Moreover, the local inhibition of mTOR‐related signalling blocks the expression of incubated cocaine‐ [19, 20] and heroin‐craving [23]. Further, acute systemic pretreatment with the FDA‐approved mTOR inhibitor Everolimus dose‐dependently reduces the magnitude of incubated cocaine‐craving in rats [21]. More recently, increased mTOR‐related signalling was also detected within the infralimbic (IL) subregion of the mPFC in female rats exhibiting incubated sucrose‐craving. Akin to cocaine‐craving rats, systemic Everolimus pretreatment blocked incubated sucrose‐craving in rats of both sexes, albeit at a higher dose than the minimally effective dose from the cocaine study (1 vs. 0.1 mg/kg) [24]. Together, these results [19, 20, 21, 22, 23, 24] suggest that increased mTOR‐related signalling within the mesocorticolimbic system may be a common biomolecular mediator of incubated craving that can be targeted by systemic treatment with FDA‐approved mTOR inhibitors.

To test this hypothesis, the present study investigated the efficacy of systemic Everolimus to reduce incubated cue‐elicited METH‐seeking in a rat model of MUD. As incubated METH‐craving is associated with mPFC dysfunction in electroencephalography studies of humans with MUD [9], we also determined whether mTOR‐related signalling within mPFC subregions is a biocorrelate of incubated METH‐craving and examined also for changes in other proteins associated with incubated cocaine‐craving, including Group 1 metabotropic glutamate receptors (mGlu1 and mGlu5) and their Homer scaffolding proteins [20, 21, 22, 25, 26].

## 2 Methods

### 2.1 Subjects

Adult female (226–250 g) and male (251–275 g) Sprague–Dawley rats were obtained from Charles River Laboratories (Hollister, CA) and housed under a 12‐h reverse light–dark cycle, with food and water available *ad libitum*. Rats were handled daily for 1 week before the experimental procedures began. All procedures adhered to the National Institutes of Health guidelines outlined in the *Guide for the Care and Use of Laboratory Animals* and were approved by the Animal Care and Use Committee at the University of California, Santa Barbara.

### 2.2 Methamphetamine Self‐Administration and Test for Incubated Craving

Detailed in the Supporting Information Methods, the surgical and operant‐conditioning procedures, as well as those employed to determine estrous cycle phase, were similar to those described in our recent studies of incubated cocaine‐craving [27, 28], with the exception that the reinforcer was a 0.1 mg/0.1 mL infusion of METH (e.g., [29]). To determine METH‐related changes in protein expression during early versus later withdrawal and to control for any time‐dependent changes in protein expression unrelated to METH experience, two additional groups of rats were included in our study. As detailed in the Supporting Information Methods, these SHAM rats underwent a sham IV surgery, followed by 10‐days exposure to the operant chambers in which responding on the active lever resulted in the presentation of the light‐tone compound stimuli in the absence of any METH delivery.

**FIGURE 1:**
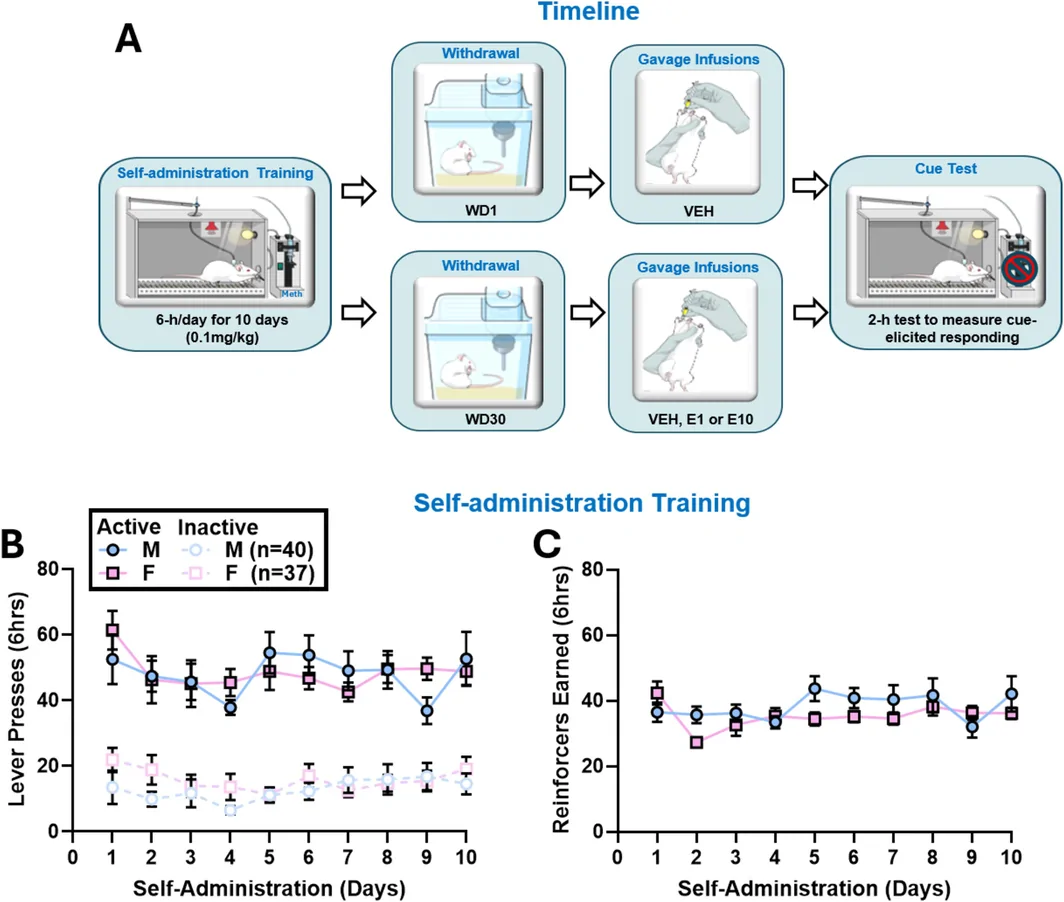
(A) Time‐line of experimental procedures to determine the effects of 1 or 10 mg/kg Everolimus on incubated METH‐craving. Comparison of the number of active and inactive lever‐presses (B) and the number of reinforcers earned (C) by male and female rats over the course of the 10‐day self‐administration phase of the study. The data represent the means ± SEMs of the individual rats indicated.

To gauge the magnitude of incubated METH‐craving, one METH and one SHAM control group was gavage‐infused with vehicle (VEH; 1% DMSO in water; vol: 1 mL/kg) prior to testing on withdrawal Day 1 (WD1), whereas another METH and SHAM control group was gavage‐infused with VEH prior to testing on withdrawal Day 30 (WD30). To examine the effects of mTOR inhibition on the expression of the incubated response, two additional groups of METH rats were gavage‐infused with either 1 or 10 mg/kg of the mTOR inhibitor Everolimus (respectively, E1 and E10; Tocris Bioscience, Minneapolis, MN). This between‐subjects approach to demonstrate the expression of incubated METH craving is as follows: 1) consistent with our prior studies of incubated craving [20, 21, 22, 25, 26, 27, 28]; 2) preferred over a within‐subjects design as it avoids any interpretational confounds related to extinction learning that might occur during testing on WD1; and 3) necessitated by brain tissue collection following testing on WD1. A time‐line of procedures is provided in Figure 1A. Study originally commenced by determining the effects of 1 mg/kg Everolimus—a dose that effectively blocks both incubated cocaine‐ [21] and sucrose‐craving [24]. However, null results with 1 mg/kg Everolimus then prompted a test of the effects of 10 mg/kg Everolimus—a dose 100‐fold higher than the minimally effective dose (0.1 mg/kg) for blocking incubated cocaine‐craving [21].

Thirty minutes post‐infusion [21, 24], both SHAM and METH rats underwent a 2‐h cue test during which pressing on the right/active lever resulted in the presentation of the light‐tone compound stimulus only, whereas pressing on the left/inactive lever had no programmed consequences.

### 2.3 Immunoblotting

Immediately after completion of cue testing, all rats were rapidly decapitated and the IL and prelimbic (PL) subregions of the mPFC dissected out over ice to determine the biocorrelates of METH‐craving on WD1 and its incubation with protracted withdrawal (Experiment 1) and to confirm an effect of Everolimus on mTOR activation (Experiments 2 and 3). To determine the relationship between METH‐craving and protein expression, Experiment 1 compared protein expression within the PL and IL tissue from both SHAM‐VEH and METH‐VEH rats following their tests for craving on WD1 versus WD30 and rationale for and details of, the electrophoresis gel‐loading procedures are provided in the Supporting Information Methods. In breif, as the 15‐lane electrophoresis gels can only accommodate *n* = 3–4 rats/experimental group, the tissue from male and female rats were processed separately and the sample sizes for the METH rats in Experiment 1 were capped at *n* = 10/group to ensure equal representation of all four experimental groups across our electrophoresis gels. For experimental groups with sample sizes greater than 10, only the first 10 rats from that group underwent immunoblotting in Experiment 1 (i.e., not all rats tested for behaviour underwent immunoblotting procedures in Experiment 1). A second immunoblotting study then determined the efficacy of Everolimus to alter indices of Akt and mTOR activation in METH‐incubated rats by comparing tissue from WD30‐VEH, WD30‐E1 and WD30‐E10 METH rats. For Experiment 2, the tissue from all rats could be accommodated by our electrophoresis gels and therefore, immunoblotting was conducted on the tissue from all rats from these three treatment groups. Evidence from the cancer literature indicates that the therapeutic efficacy of mTOR inhibitors and/or the magnitude of their effects upon intracellular signalling varies as a function of baseline Akt/mTOR activation, with inhibitor sensitivity correlated positively with basal kinase activation [30, 31]. The null results from our first two immunoblotting studies (see below) prompted a third follow‐up immunoblotting study examining the effects of the 10 mg/kg Everolimus dose on basal phospho‐kinase expression in experimentally naive rats. For this, we compared protein expression from the tissue of WD1‐SHAM controls who were gavage‐infused with VEH and an additional group of METH‐naive rats gavage‐infused with 10 mg/kg Everolimus (Experiment 3). The specific procedures for tissue dissection, homogenization and immunoblotting for all these studies are detailed in the Supporting Information Methods.

### 2.4 Statistical Analyses

Both the behavioural and immunoblotting results were analysed using analyses of variance (ANOVAs), with α set to 0.05 and correlational analyses between behaviour and protein expression were conducted using Pearson correlation tests. The details of the statistical analyses are also provided in the Supporting Information Methods.

## 3 Results

### 3.1 Methamphetamine Reinforcement

An analysis of the time‐course of behaviour during the self‐administration phase of the study revealed a significant Group × Sex × Day interaction for the number of active lever‐presses [F(27,621) = 1.563, *p* < 0.036] and deconstruction of this interaction along the Day factor further revealed a main Group effect on Day 1 [F(3,76) = 2.186, *p* < 0.05] and Day 8 [F(3,76) = 3.098, *p* < 0.05], as well as a strong trend for a Sex × Group interaction on Day 8 [F(3,76) = 3.697, *p* < 0.05l]. On Day 1, rats slated for WD30‐VEH pressed significantly more on the active lever compared to the WD1‐VEH group (Tukey post hoc tests: *p* = 0.036). On Day 8, the Sex × Group interaction reflected a group difference in males [F(3,39) = 5.190, *p* < 0.05] but not females (*p* = 0.486), that reflected higher responding by males slated for WD30‐E10 versus all of the other groups (Tukey post hoc: all *p* < 0.05). As these group differences were not consistent over the course of the self‐administration phase of the study, the data for active lever‐responding is presented as the average of the three pretreatment groups, separately for male and female rats in Figure 1B. As also apparent in Figure 1B, no main effects or interactions were detected with respect to the time‐course of inactive lever‐pressing (Group × Sex × Day ANOVA, all *p* > 0.243). We also detected a main Day effect for the number of reinforcers earned [F(9,621) = 2.395, *p* < 0.05], but there were no main effects of, or interactions with, either the Group or Sex factors for this variable (Figure 1C; all *p* > 0.159). Further, we observed no overt effect of estrous phase on the behaviour of female rats during this phase of the study, although statistical analyses were not performed due to small sample sizes for certain estrous phases (Figure S1). Together, these data for the self‐administration phase of the study indicate no consistent group, sex or estrous phase differences in behaviour prior to cue‐testing.

The behavioural results for SHAM controls employed in immunoblotting are summarized in the Supporting Information Results (see Table S1).

### 3.2 Everolimus Effects on Incubated Methamphetamine‐Seeking

**FIGURE 2:**
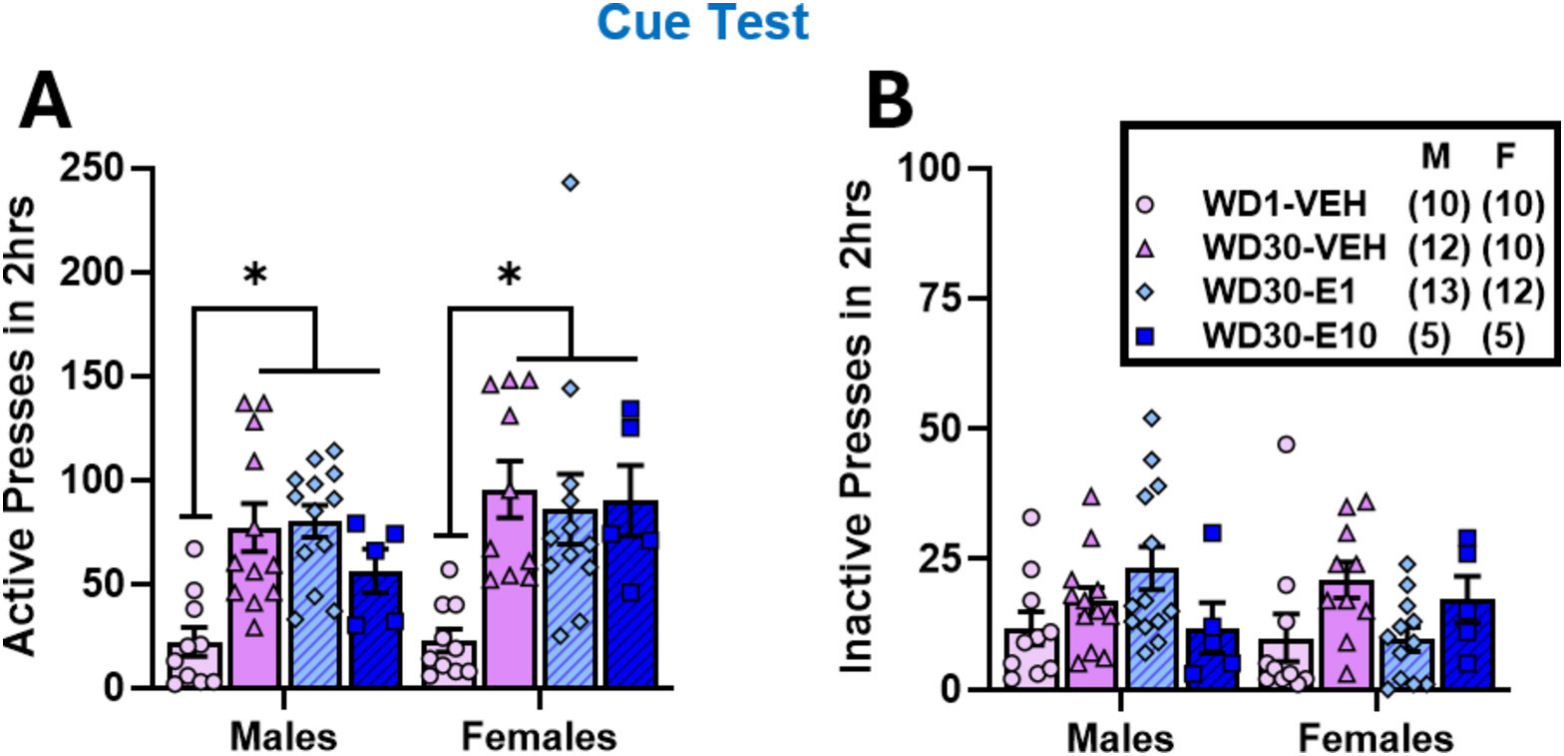
Comparison of cue‐reinforced behaviour exhibited by male and female rats during the test for cue‐elicited METH‐seeking. The average number of active (A) and inactive (B) lever‐responses during a 2‐h test of cue‐reinforced responding emitted by male and female rats. The data represent the means ± SEMs of the individual rats indicated. *p < 0.05 WD1 vs. WD30 (Withdrawal effect).

Inspection of Figure 2A suggested that the 10 mg/kg Everolimus may have lowered responding in males. However, no main Sex effect or Group × Sex interaction were detected for the number of active lever‐presses exhibited during the Cue Test (Sex effect and interaction: *p* > 0.108). This null result precluded tests for sex‐specific post hoc comparisons and the data were collapsed across the Sex factor for an examination of Everolimus' effects on the expression and magnitude of incubated METH‐craving. This analysis indicated a significant Group effect for the number of cue‐reinforced active lever‐presses [F(3,76) = 13.142, *p* < 0.001], that reflected more active lever‐pressing in the 3 groups tested on WD30 versus WD1‐VEH controls, indicative of incubated METH‐craving (Figure 2A; Tukey's tests, all *p* < 0.005). Further, relative to WD30‐VEH controls, neither Everolimus dose significantly lowered the magnitude of responding on WD30 (Tukey's tests, all *p* > 0.820). Thus, Everolimus did not affect the expression or magnitude of incubated METH‐craving. A Sex by Group interaction was detected for the number of inactive lever‐presses on the Cue Test (Figure 2B) [F(3,76) = 2.995, *p* = 0.037; Sex and Group effects: *p* > 0.144]. However, deconstruction of this interaction along the Sex variable did not reveal any specific group differences in rats of either sex (one‐way ANOVAs, males: *p* = 0.70; females: *p* = 0.103). Finally, we observed no overt effect of estrous cycle phase on active or inactive lever‐responding during the Cue Test, although the small sample sizes preluded analyses of these data (see Supporting Information Results).

### 3.3 Biocorrelates of Incubated Methamphetamine‐Seeking

#### 3.3.1 mTOR and Akt

**FIGURE 3:**
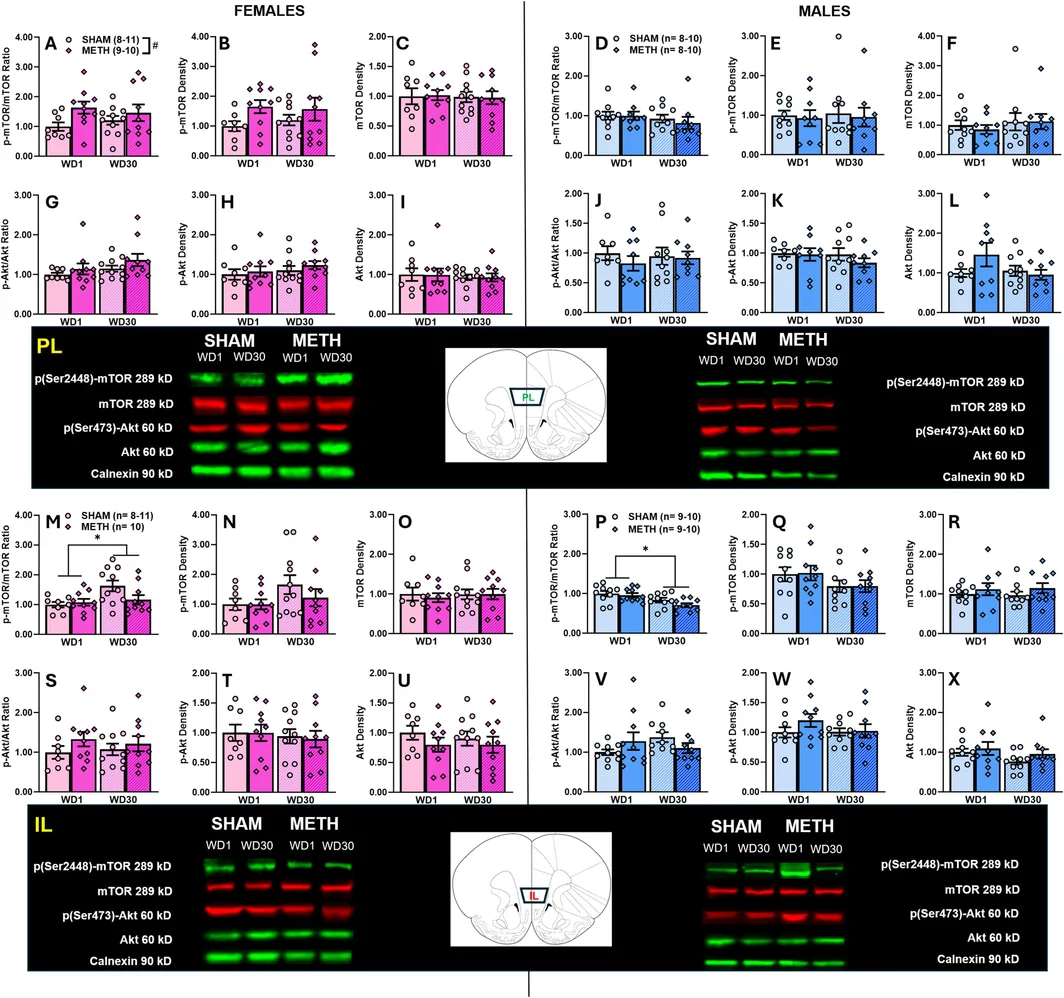
Immunoblotting results for mTOR and Akt in the PL and IL for female and male SHAM vs. METH‐experienced rats. Data from the PL (Female, Male, respectively): (A,D) p (Ser2448)‐mTOR:mTOR, (B,E) p (Ser2448)‐mTOR, (C,F) total mTOR, (G,J) p (Ser473)‐Akt:Akt, (H,K) p (Ser473)‐Akt, (I,L) total Akt. Data from the IL (Female, Male, respectively): (M,P) p (Ser2448)‐mTOR:mTOR, (N,Q) p (Ser2448)‐mTOR, (O,R) total mTOR, (S,V) p (Ser473)‐Akt:Akt, (T,W) p (Ser473)‐Akt, (U,X) total Akt. The data are expressed as a fold‐change relative to WD1‐SHAM controls and represent the means ± SEMs of the individual rats indicated (*n* = 8–11). A cartoon depicting the tissue PL and IL dissection and representative immunoblots are also provided. Group differences are indicated in relevant panels by #*p* < 0.05 SHAM vs. METH (METH history effect); *p < 0.05 WD1 vs. WD30 (Withdrawal effect).

Given the negative behavioural results for Everolimus (Figure 2), we next determined whether incubated METH‐seeking is related to the expression and/or activational state of Akt/mTOR‐related signalling within mPFC subregions. A Drug × Withdrawal ANOVA indicated relative p (Ser2448)‐mTOR expression differed within female PL with METH‐experienced rats exhibiting higher levels of relative p‐mTOR levels [Drug effect: F(1,38) = 4.691, *p* < 0.05], irrespective of withdrawal (Figure 3A). No other group differences for our indices of mTOR (Figure 3B–C, all *p* > 0.057) or Akt (Figure 3G–I, all *p* > 0.120) expression/activation were observed within female PL, nor were there differences for mTOR (Figure 3D–F; all *p* > 0.275) or Akt (Figure 3J–L; all *p* > 0.133) expression/activation within male PL. Although METH‐seeking was not correlated with our measures of mTOR expression/activation within the PL of neither male nor female rats (Table S2), relative p (Ser473)‐Akt expression within the PL was positively correlated with the magnitude of METH‐seeking but in female rats only (see Figure S2A).

Within the female IL, WD30 rats exhibited higher relative p‐mTOR levels, regardless of their METH history [Withdrawal effect: F(1,38) = 5.782, *p* < 0.05]—an effect that appeared to be driven by the WD30 SHAM controls and not the METH‐incubated rats (Figure 3M). METH‐experienced females also did not differ from their SHAM controls regarding our other indices of mTOR expression/activation within the IL (Figure 3N,O; Drug × Withdrawal ANOVAs: for both p (Ser2448)‐mTOR and mTOR, all *p* > 0.100) nor were our indices of mTOR or Akt expression/activation within the IL correlated with METH‐seeking behaviour in female rats, irrespective of the inclusion of the outlier WD30‐VEH control (Figure S2B; Table S2). In male IL, a time‐dependent decrease in relative p (Ser2448)‐mTOR expression was observed, irrespective of METH history (Figure 3P) [Withdrawal effect: F(1,39) = 12.844, *p* < 0.001; other *p* > 0.443]. Although no METH‐related changes in total mTOR expression or phosphorylation were detected in male IL (Figure 3Q,R; Drug × Withdrawal ANOVAs: for both p (Ser2448)‐mTOR and mTOR, all *p* > 0.064), relative p (Ser2448)‐mTOR expression within the male IL was inversely correlated with METH‐seeking behaviour (r = −0.511, *p* = 0.0211; Figure S2B). Finally, no group differences were detected for our measures of Akt expression or activation within the IL of either females (Figure 3S–U; Drug × Withdrawal ANOVA, all *p* > 0.175) or males (Figure 3V–X; all *p* > 0.066) nor were these measures correlated with METH‐seeking behaviour in rats of either sex (Table S2).

These results pertaining to mTOR and Akt expression/activation within vmPFC subregions of METH‐craving rats differ from our prior results for incubated cocaine‐craving in which an incubation‐specific increase in both mTOR and Akt activation was observed selectively within the PL of both male and female rats [21, 22]. The present results also differ from those reported for incubated sucrose‐craving in which an incubation‐specific reduction in Akt activation was observed within the female PL, whereas males exhibited elevated indices of both Akt and mTOR activation within the IL [24].

#### 3.3.2 Group1 mGlu Receptors and Homer Proteins

**FIGURE 4:**
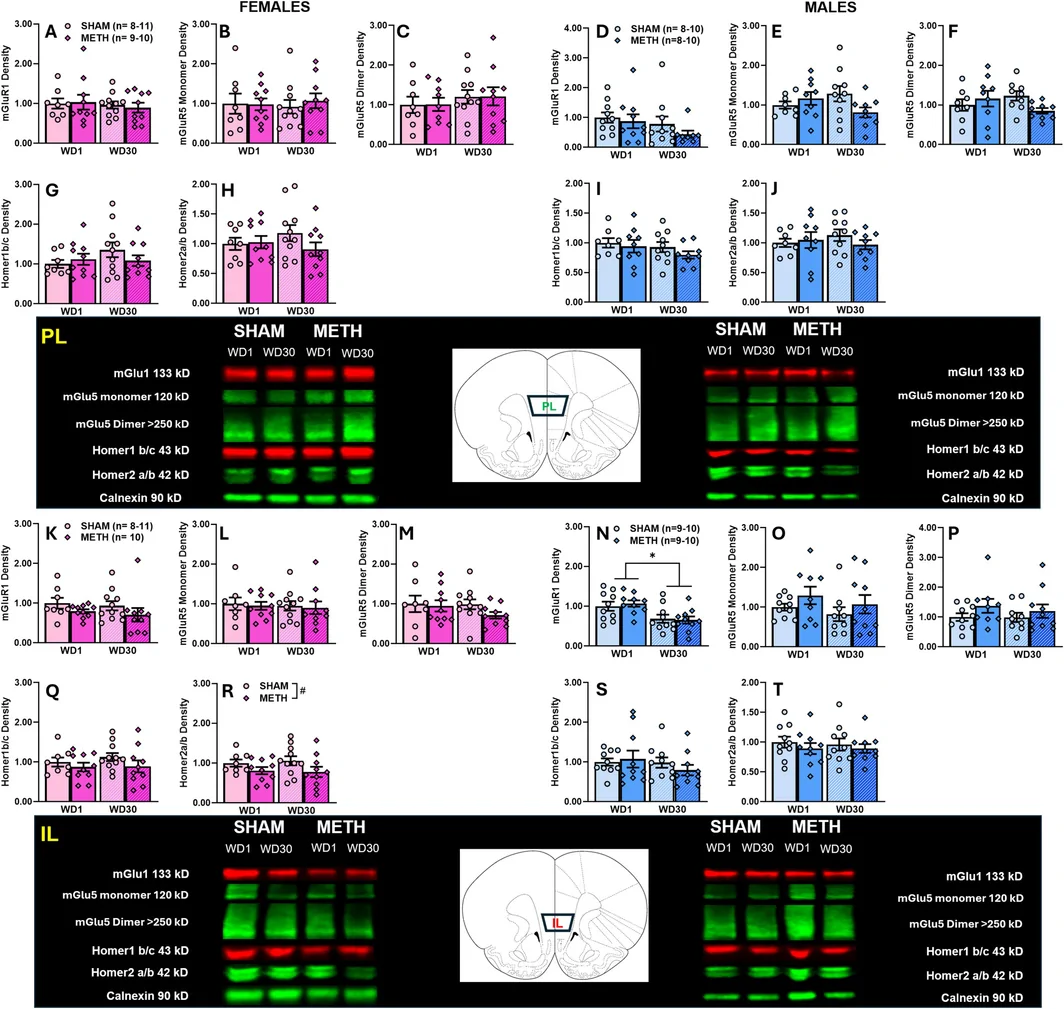
Immunoblotting results for mGlu1/5 and Homer proteins in the PL and IL for female and male SHAM vs. METH‐experienced rats. Data from the PL (Female, Male, respectively): (A,D) mGlu1, (B,E) mGlu5 monomer, (C,F) mGlu5 dimer, (G,I) Homer1b/c, (H,J) Homer2a/b. Data from the IL (Female, Male, respectively): (K,N) mGlu1, (L,O) mGlu5 monomer, (M,P) mGlu5 dimer, (Q,S) Homer1b/c, (R,T) Homer2a/b. The data are expressed as the fold‐change relative to WD1‐SHAM controls and represent the means ± SEMs of the individual rats indicated (*n* = 8–11). A cartoon depicting the tissue PL and IL dissection and representative immunoblots are also provided. Group differences are indicated in relevant panels by #*p* < 0.05 SHAM vs. METH (METH history effect); *p < 0.05 WD1 vs. WD30 (Withdrawal effect).

Incubated cocaine‐ [21, 22, 25, 26] and sucrose‐craving [24] is associated with reinforcer‐specific changes in mGlu1, mGlu5 and their Homer scaffolding proteins within mPFC subregions and so we examined their relation to incubated METH‐craving. No group differences were detected for the expression of Group 1 mGlu receptors or either Homer protein in female PL (Figure 4A–C,G,H) [Drug × Withdrawal ANOVAs, all *p* > 0.207], although Homer2a/b expression in this subregion was inversely correlated with METH‐seeking (Figure S2C). Within the male PL, a Drug × Withdrawal interaction was detected for the mGlu5 monomer (Figure 4E) [F(1,35) = 4.30, *p* = 0.046; other *p* > 0.329]. However, deconstruction of this interaction along the Drug factor revealed no time‐dependent changes in either SHAM controls (*p* = 0.233) or METH‐experienced rats (*p* = 0.104). No other differences in mGlu receptor or Homer protein expression were detected in male PL (Figure 4D–F,I,J; Drug × Withdrawal ANOVAs, all *p* > 0.064) nor was the expression of mGlu receptors or Homer proteins in this subregion correlated with METH‐seeking behaviour in males (Table S2).

In contrast to the PL, METH‐experienced females expressed lower Homer2a/b in the IL overall, compared to SHAM controls (Figure 4R) [Drug effect: F(1,38) = 4.489, *p* = 0.041; other *p* > 0.682], with no METH‐SHAM differences observed for mGlu1, mGlu5 monomer/dimer or Homer1b/c expression within female IL (Figure 4K–Q; Drug × Withdrawal ANOVAs, all *p* > 0.095). Of these proteins, only mGlu1 expression was correlated with METH‐seeking in female rats (Figure S2C,C′), the direction of the relationship varied with the inclusion versus exclusion of the WD30‐VEH female outlier that pulled the relation in a positive direction owing to her high level of responding. In male IL, mGlu1 levels were lower on WD30, irrespective of METH experience [Figure 4N; F(1,39) = 14.190, *p* < 0.001; other *p* > 0.604] and mGlu1 expression within this subregion was inversely correlated with METH‐seeking in male rats (r = −0.536, *p* = 0.015; Figure S2D). No other group differences were detected for the other proteins examined (Figure 3O,P,S,T) (Drug × Withdrawal ANOVAs, all *p* > 0.132) nor were any other significant correlations observed within male IL (Table S2).

Although we have yet to immunoblot for Group 1 mGlu receptors and Homer expression within the vmPFC of cocaine‐incubated female rats, the present results for these proteins within vmPFC subregions of METH‐craving rats also differ from our prior results for cocaine‐craving males in which an incubation‐specific reduction in both mGlu1 and mGlu5 [21, 22, 25], as well as an increase in Homer2a/b [21, 22, 26], are detected within the PL or more ventral aspect of the PFC. Likewise, the present results for METH‐craving differ also from our prior results of incubated sucrose‐craving in which mGlu5 expression is lower within the female PL, but higher in the male PL and both mGlu5 and mGlu1 expressions are higher in the male IL, with no changes in Homer proteins observed for either sex within either vmPFC subregion [24].

### 3.4 Everolimus Effects on Akt/mTOR Signalling Within vmPFC Subregions

**FIGURE 5:**
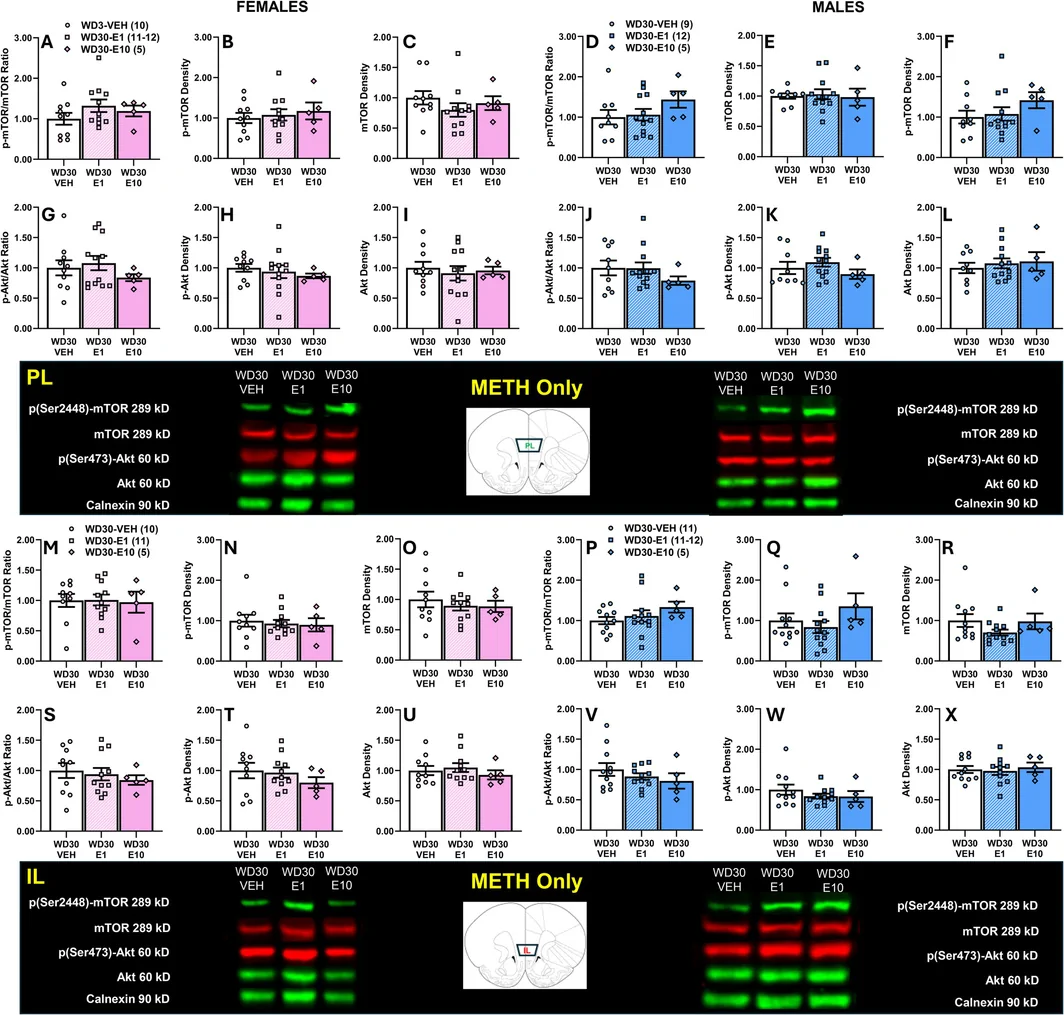
Immunoblotting results for the effects of Everolimus pretreatment on mTOR and Akt expression within the PL and IL of METH‐experienced female and male rats. Data from the PL (Female, Male, respectively): (A,D) p (Ser2448)‐mTOR:mTOR, (B,E) p (Ser2448)‐mTOR, (C,F) total mTOR, (G,J) p (Ser473)‐Akt:Akt, (H,K) p (Ser473)‐Akt, (I,L) total Akt. Data from the IL (Female, Male, respectively): (M,P) p (Ser2448)‐mTOR:mTOR, (N,Q) p (Ser2448)‐mTOR, (O,R) total mTOR, (S,V) p (Ser473)‐Akt:Akt, (T,W) p (Ser473)‐Akt, (U,X) total Akt. The data are expressed as the fold‐change relative to WD30‐VEH controls and represent the means ± SEMs of the individual rats indicated (*n* = 5–12). A cartoon depicting the tissue PL and IL dissection and representative immunoblots are also provided. No group differences were detected.

We next determined whether systemic Everolimus effectively reduced Akt/mTOR activation within mPFC subregions of rats tested on WD30. However, we detected no Everolimus effect on the total or relative expression of mTOR, Akt, p (Ser2448)‐mTOR or p (Ser473)‐Akt (Figure 5) (Pretreatment effects: for females, *p* > 0.295; for males, *p* > 0.200). Thus, systemic Everolimus pretreatment did not lower the activational state of either Akt or mTOR within the PL or IL subregions of METH‐experienced rats. As the possibility existed that these null Everolimus results might reflect the fact that basal kinase activation within vmPFC was unaffected by METH experience and withdrawal (Figure 3), we then examined the effects of 10 mg/kg Everolimus on indices of Akt/mTOR activation within the PL and IL of METH‐naive male rats. As depicted in Figure S3, we failed to detect any changes in the total or relative expression of p (Ser473)‐Akt or p (Ser2448)–mTOR in either subregion of METH‐naive rats (Pretreatment effects: *p* > 0.129).

## 4 Discussion

The pharmacological efficacy of PI3K/Akt/mTOR inhibitors at blocking the expression of incubated craving for cocaine [19, 20, 21], heroin [23] and sucrose [24] prompted our investigation into the functional relevance of mTOR for incubated METH‐craving. Herein, an extended‐access model of METH self‐administration was used to test the effects of the orally bioavailable mTOR inhibitor, Everolimus, on cue‐induced METH‐craving, alongside immunoblotting analyses to examine changes in PL and IL mTOR signalling during cue‐elicited craving. Behavioural results confirmed incubated METH‐craving, as evidenced by significantly higher cue‐reinforced responding in the VEH‐infused groups tested on WD30 versus WD1 (Figure 2A). However, neither Everolimus dose (1 and 10 mg/kg) reduced the magnitude of incubated cue‐induced METH‐seeking, with both Everolimus pretreatment groups expressing incubated craving, relative to WD1‐VEH cotrols (Figure 2A). This result was unexpected given that Everolimus completely blocks incubated cocaine‐seeking at doses as low as 0.1 mg/kg [21]. As our studies of incubated cocaine‐ [21], sucrose‐ [24] and METH‐craving (present study) are the first to examine the effects of acute oral pretreatment with Everolimus on cue‐elicited craving, the possibility exists that the Everolimus dose‐craving function may exhibit a U‐shape, with only lower doses effective at reducing incubated craving. Until the present METH study, we had not tested the effects of Everolimus doses > 1 mg/kg as this dose effectively blocked the expression of both incubated cocaine‐ [21] and sucrose‐seeking [24], albeit with a larger effect size in the case of cocaine. Moreover, 0.1 and 1.0 mg/kg Everolimus are equipotent at completely blocking incubated cocaine‐craving, whereas the cocaine‐craving of rats pretreated with 0.01 mg/kg Everolimus is intermediate that of 0.1 mg/kg Everolimus and VEH controls [21]. Thus, at least in cocaine‐incubated rats, the Everolimus dose‐craving function appears to be a step‐wise, descending, function in which maximum inhibition of incubated craving is observed at 0.1 mg/kg, with no evidence of a waning of the drug effect the 10‐fold higher dose of 1 mg/kg [21]. Although it is possible that a history of METH‐taking might shift the Everolimus dose‐craving function to the right of cocaine‐ and/or sucrose‐experienced rats, solubility issues precluded the study of higher doses (e.g., 100–1000 mg/kg). Nevertheless, the fact that Everolimus doses 10 to 100‐fold higher than those maximally effective at reducing incubated cocaine‐craving failed to impact incubated METH‐seeking argues that time‐dependent changes in cue‐elicited METH‐seeking do not likely involve the Akt/mTOR signal transduction pathway.

Supporting this conclusion, we detected no METH incubation‐specific changes in indices of mTOR or Akt activation within the PL or IL of rats of either sex (Figure 3). As yet determined in females, cue‐elicited cocaine‐craving is positively correlated with PI3K/Akt/mTOR activation within both the PL and the IL of male rats [20, 21]. Herein, two relationships between the magnitude of cue‐elicited METH‐craving and indices of mTOR/Akt activation were detected. Specifically, METH‐craving was positively correlated with Akt activation within the female PL, whereas METH‐craving was negatively correlated with mTOR activation within the male IL (Figure S2). Whether or not the apparent sex differences in Akt activation (or any other protein change or correlate discussed below) reflect a genuine sex difference in protein expression cannot be deciphered from the design of our immunoblotting studies as male and female tissue was not compared directly and we had insufficient tissue to conduct follow‐up comparisons between sexes to confirm the presence/absence of a sex difference in protein expression. Nevertheless, as reported in other studies of incubated METH‐craving [18], we detected no sex differences in METH reinforcement, intake or cue‐elicited METH‐seeking in this study (Figures 1, 2). Although the behavioural relevance of what appears to be sex‐selective correlations is not clear at present, these correlations further the argument that the magnitude of METH‐craving is dissociable from our measures of kinase activation within mPFC subregions.

Curiously, Everolimus pretreatment did not reduce indices of Akt/mTOR activation in either vmPFC subregion of METH‐experienced rats (Figure 4A–L), indicating no suppression of Akt/mTOR signalling by doses as high as 10 mg/kg (Figure 4A–L). This contrasts with our prior results for pretreatment with 1 mg/kg Everolimus in the context of both incubated cocaine‐ [21] and sucrose‐craving [24]. Notably, in both the cases of incubated cocaine‐ and sucrose‐craving, p‐mTOR expression is higher within the PL and IL, respectively, of rats exhibiting incubated craving, relative to rats tested in early withdrawal (i.e., cue‐induced p‐mTOR expression incubated during abstinence) [21, 24]. Given our null immunoblotting results for both METH and Everolimus, we hypothesized that the lack of an Everolimus effect in the present METH study relates to the fact that mTOR was not activated by METH‐associated cues in the first place (Figure 3). Indeed, evidence from the cancer literature indicates that basal Akt/mTOR activation is required for sensitivity to the therapeutic and intracellular signalling effects of rapamycin and its derivatives, including Everolimus [30, 31]. Consistent with this, the 10 mg/kg Everolimus dose did not impact phospho‐kinase expression within the vmPFC of METH‐naive rats (Figure S3). Collectively, our results to date suggest that Everolimus attenuates incubated craving only when Akt/mTOR signalling is hyper‐active within at least one vmPFC subregion, arguing against the therapeutic potential of Everolimus or other mTOR inhibitors as a METH‐craving intervention.

In male rats, incubated cocaine‐craving is also associated with lower Group 1 mGlu receptor within the PL/ventral aspect of the mPFC [21, 22, 25] and the magnitude of cue‐elicited cocaine‐craving by males is inversely related to both mGlu1 and mGlu5 expression within this subregion [21, 25]. Herein, we detected no incubation‐specific changes in the vmPFC expression of either mGlu receptor in rats of either sex (Figure 4). However, the magnitude of METH‐craving was inversely correlated with mGlu1 levels within male IL (Figure S2C), highlighting the potential for low vmPFC mGlu1 expression as a common biocorrelate of incubated cocaine‐ and METH‐craving. In the context of incubated cocaine‐craving, we theorize that reduced vmPFC mGlu1 expression reflects receptor desensitization, secondary to augmented cue‐elicited glutamate release within this subregion [30]. Although it remains to be determined whether incubated METH‐craving is associated with enhanced cue‐elicited glutamate release within vmPFC to possibly account for lower mGlu1 expression within the IL, re‐exposure to METH‐associated cues elicits a robust rise in extracellular glutamate within the PL of rats tested under extinction‐reinstatement procedures [31], supporting this possibility. However, neither mimicking nor stimulating low vmPFC mGlu1 expression impacts the magnitude of incubated cocaine‐craving; rather, this neuroadaptation appears to impair extinction learning, maintaining persistently high levels of cocaine‐seeking behaviour upon repeated cue re‐exposure during protracted withdrawal [25]. As IL mGlu1 expression was inversely correlated with the magnitude of cue‐elicited METH craving, at least in male rats (Figure S2C), an important goal of future work is to determine the functional relevance of low IL mGlu1 expression for both the magnitude and persistence of incubated METH‐craving and potential sex differences therein.

Irrespective of the duration of METH withdrawal, female rats exhibited lower IL expression of the glutamate receptor scaffolding protein Homer2a/b, relative to their SHAM controls (Figure 3R). Although these same females did not exhibit any overt change in mGlu1 or mGlu5 expression, this finding raises the possibility that incubated METH‐craving might relate to impaired glutamate‐dependent signalling consequent to downregulated scaffolding of mGlu1/5 or other Homer‐interacting partners within the female IL (e.g., NMDA receptor/PSD95). It is noteworthy that all METH‐related protein changes and correlations between METH‐craving and protein expression detected herein were sex‐selective, despite no sex differences in, or overt effects of estrous cycle on, MUD‐related behaviour. Although neither mGlu1 nor Homer2a/b is associated with incubated sucrose‐craving in either sex, marked sex and subregional differences exist for mGlu5, Akt/mTOR activation and PKC epsilon expression within vmPFC, in the face of comparable levels of incubated sucrose‐craving between male and female rats [24]. Whether such sex differences reflect sex‐selective priming of specific receptors/intracellular signalling pathways within mPFC remains to be vetted experimentally. However, in contrast to both incubated METH‐ and sucrose‐craving, incubated cocaine‐craving is associated with increased PL Homer2a/b expression, at least in male rats [21, 22, 26]. Thus, distinctions exist with respect to the direction of effect, the subregion, the sex‐selectivity and the Homer isoform associated with incubated METH‐ versus both cocaine‐ and sucrose‐craving that argue against Homer proteins as common regulators of incubated craving generally. Furthering this notion, reversing the apparent imbalance in Homer2a/b vs. Homer1b/c expression within the PL of cocaine‐incubated rats does not alter the magnitude nor rate of extinction of cue‐elicited cocaine‐seeking when rats are tested in a drug‐free state, but it does prevent cocaine‐primed reinstatement of cocaine‐seeking behaviour [26]. Thus, drug‐specific changes in mPFC Homer expression may regulate more so drug‐induced rather than cue‐induced relapse‐like behaviour.

METH and cocaine exert their psychomotor stimulant effects via different mechanisms; cocaine inhibits the reuptake of monoamine neurotransmitters by blocking plasma membrane transporters (c.f., [32]), whereas the pharmacology of METH is more complex, serving as a substrate for plasma membrane and vesicular monoamine transporters, inhibiting monoamine oxidase (e.g., [33, 34]), acting as a agonist at trace amine‐associated receptor 1 (TAAR1) (e.g., [35]) and an antagonist at NMDA‐type glutamate receptors in a manner independent of direct channel inhibition (e.g., [36]). Given this, perhaps it is not surprising that divergent biomolecular adaptations within mPFC [21, 22, 25, 26], present study] and its major efferent target, the nucleus accumbens [16, 17], are associated with incubated craving for these two stimulant drugs. The possibility also exists that incubated cocaine‐ and METH‐craving involve the activation of distinct neural circuits. Indeed, transient inactivation of mPFC subregions is ineffective at blocking the expression of incubated METH‐craving [11], whereas pharmacological inhibition of mPFC subregions (the PL in particular) consistently blunts incubated cocaine‐seeking [20, 27, 28, 37, 38, 39]. Thus, it is likely that adaptations in many regions collaborate to drive incubation and reversing just a subset may be insufficient to short‐circuit incubation. This being said, the expression of both incubated cocaine‐ and METH‐craving involve the strengthening of excitatory synapses within the core subregion of the nucleus accumbens that require the insertion of calcium‐permeable AMPA receptors [16, 18, 40]. Incubated METH‐craving can persist for several months in individuals with MUD, rendering them highly vulnerable to cue‐induced relapse [8, 9]. Given the cocaine‐centric nature of the incubation of craving literature (c.f. [41]), the biochemical and pharmacological results of the present study, coupled with those of prior studies of incubated METH‐craving [11, 12, 14, 16, 17, 18], highlight the importance of identifying common and reinforcer‐specific neuroadaptations that drive incubated craving of relevance to the effective targeting of this clincially relevant phenomenon, as well as the generalizability and/or off‐target profiles of potential anti‐craving medications for treating MUD.

## Author Contributions

Conceptualization: FJC, TEK, KKS; Methodology: FJC, LLHS; TEK, KKS; Formal analysis: FJC, LLHS, KKS; Investigation: FJC, KES, KS, AJ, EL, HMN, SDA, LLHS, CJED, SMD, CAI; Resources: TEK, KKS; Data Curation: FJC,LLHS, KKS; Writing – Original Drafts: FJC, KES, KS, LLSH, SDA, SMD, CAI; Writing – Review and Editing: FJC, TEK, KKS; Visualization: FJC, LLHS, KKS; Supervision: FJC, LLHS, TEK, KKS; Project Management: TEK, KKS; Funding Acquisition: FJC, KES, SMD, SDA, TEK, KKS.

## Funding

This work was supported by the National Institute on Drug Abuse (10.13039/100000026; DA053328; DA051100; DA063695), National Institute of Neurological Disorders and Stroke (10.13039/100000065; NS141388), Bill and Melinda Gates Foundation (10.13039/100000865), National Science Foundation (10.13039/100000001), and NSF‐AGEP CA HSI Alliance Fellowship.

## Ethics Statement

We confirm that this original work adhered to ethical standards and regulations. All authors significantly contributed to and approved the final version of this report.

All procedures adhered to the National Institutes of Health guidelines outlined in the *Guide for the Care and Use of Laboratory Animals* and were approved by the Animal Care and Use Committee at the University of California, Santa Barbara.

## Conflicts of Interest

The authors declare no conflicts of interest.

## Supporting information




**Data S1:** Supporting Information.
**Table S1:** Comparison of the average number of active and inactive lever‐presses by male and female SHAM rats during the last 3 days of lever training and non‐reinforced cue testing (Cue Test). The data represent the means ± SEMs of the number of animals indicated in parentheses.
**Table S2:** Correlations between cue‐elicited METH‐seeking and protein expression within the PL and the IL of male and female rats. Sample sizes are indicated in parentheses. Significant correlations indicated by an asterisk and bolded font. mGlu5M and mGlu5D = monomer and dimer form of mGlu5, respectively.

**Figure S1:** Characterizing estrous phase following METH intake and craving. Over the last 5 days of the 6‐h METH self‐administration period, the estrous stage was identified for each rat to determine potential influences in (A) active and (B) inactive lever presses, as well as (C) reinforcers earned. The estrous phase of each rat was also identified following the 2‐h cue test. No group differences are detected.

**Figure S2:** Sex differences in the protein correlates of METH‐seeking. In the PL, cue‐elicited METH‐seeking was (A) positively correlated with p (Ser473)‐Akt:Akt in females. In the IL, cue‐elicited METH‐seeking was negatively correlated with (B) p (Ser2448)‐mTOR:mTOR and with (C) mGlu1 levels in males. Sample sizes are indicated in parentheses and the results from the correlational analyses are included in each panel.

**Figure S3:** Immunoblotting results for the effects of Everolimus pretreatment on mTOR and Akt expression within the PL and IL of METH‐naive male and female rats. PL (Female, Male, respectively): (A,D) p (Ser2448)‐mTOR:mTOR, (B,E) p (Ser2448)‐mTOR, (C,F) total mTOR, (G,J) p (Ser473)‐Akt:Akt, (H,K) p (Ser473)‐Akt, (I,L) total Akt. IL (Female, Male, respectively): (M,P) p (Ser2448)‐mTOR:mTOR, (N,Q) p (Ser2448)‐mTOR, (O,R) total mTOR, (S,V) p (Ser473)‐Akt:Akt, (T,W) p (Ser473)‐Akt, (U,X) total Akt. The data are expressed relative to WD30‐VEH and represent the means ± SEMs of the individual rats indicated (*n* = 5–12). A cartoon depicting the tissue PL and IL dissection is provided in Figures 3, 4, 5 of the main text and representative immunoblots from this experiment are provided. No group differences were detected.

## Data Availability

The data related to this project will be made available upon request.
