# Supplementary material for: Systemic Everolimus Does Not Block the Incubation of Methamphetamine‐Craving: A Dose–Response Study
Source: Addict Biol. 2026 Sep 13;31(9):e70189. doi: 10.1111/adb.70189 (PMC13571856; doi:10.1111/adb.70189)
Supplement: Supplementary file 1 [file ADB-31-e70189-s003.docx]

**SUPPLEMENTAL MATERIALS**

**SUPPLEMENTAL METHODS**

**Lever-response training.** To instigate lever-pressing behavior, rats designated for METH self-administration were first trained to respond for food reinforcement (45 mg banana-flavored sucrose pellets; BioServ, Flemington, NJ) in operant chambers equipped with a right and left lever that were housed inside sound-attenuating cubicles (Med Associates, St. Albans, VT) for two days (6-h/day) prior to surgery. Operant chambers were interfaced with a control box connected to a PC running MED-PC V software (Med Associates) to quantify lever responses and reinforcers earned. Right lever presses resulted in the presentation of a 20-s light-tone paired stimulus along with a 45 mg banana-flavored sucrose pellet (Bio Serv; Flemington, NJ, USA). Additional presses during an ongoing 20-s cue presentation did not result in further sucrose pellets. Presses on the left lever had no programmed consequences.

**Surgical Procedures.**

*Intravenous Catheterization.* Under isoflurane anesthesia (3% for induction; 1.0-2.5% for maintenance), rats designated for METH self-administration studies underwent jugular vein catheter implantation. The catheters were made from SILASTIC® tubing (10 cm, i.d. 0.012 in, o.d. 0.025 in; Dow Corning, Midland, MI), sterilized in Cidex (Neta Scientific Inc, Marlton, NJ), and rinsed with sterile saline (Medline Industries LP, Pasadena, CA) before surgery. Each catheter was secured with sterile sutures into the jugular vein through a small ventral neck incision, then tunneled subcutaneously around the right shoulder, exiting through a dorsal incision between the shoulder blades. The external end was attached to a 22-gauge nonferrous metal cannula encased in a plastic screw connector (PlasticsOne®, Roanoke, VA), which was secured onto a nylon spacer (10 mm, i.d. 3.2 mm, o.d. 5 mm; Grainger Inc, Palatine, IL) and inserted into a backpack-style harness (Instech, Plymouth Meeting, PA), following the method described by LaCrosse et al. [1]. To minimize catheter/harness damage, rats were single-housed following surgery and remained single-housed for the remainder of the study.

*Sham Surgery.* To index baseline protein expression in our immunoblotting studies, a group of male and female rats underwent a sham surgical procedure (SHAM) that involved anesthesia and a ventral neck incision, without catheter implantation or harness fitting, followed by suture closure.

*Postoperative Care.* Postoperative care was provided to all animals for four consecutive days. For the first two days, rats received subcutaneous injections of the analgesic meloxicam (2 mg/kg; Midwest Veterinary Supply Inc, Lakeview, MN). Behavior and surgical wounds were closely monitored for any abnormalities throughout the recovery period. To maintain catheter patency, daily flushing was performed using Cefazolin (100 mg/ml, 0.1 ml; Covetrus North America, Visalia, CA) and Heparinized saline (70 U/ml, 0.1 ml; Medline Industries LP), with patency confirmed weekly via intravenous infusion of Brevital (methohexital sodium, 10 mg/ml, 0.1 ml; Covetrus North America).

**Methamphetamine Self Administration.** Following 4 days of post-operative care, METH rats were trained to self-administer METH for 10 consecutive days (6 h/day). The operant-conditioning procedures for METH self-administration were similar to those described for lever-response training, except that right lever presses now delivered intravenous METH (0.1 mg/kg/0.1 ml infusion), which was paired with the 20-s light + tone compounded stimulus. On the first day of METH self-administration, the maximum number of METH reinforcers was capped at 100 infusions to prevent accidental overdose. Again, responses on the left lever yielded no programmed consequences. During the METH self-administration phase of the study, SHAM rats were placed into their respective operant chambers for 6 h/day and right lever-presses resulted in the presentation of the tone-light stimulus, while left lever-presses had no programmed consequences. As the SHAM rats were not fitted with an intravenous catheter, they did not receive any METH infusions during any phase of the study. After the 10-day self-administration period, all rats were left undisturbed in the home cage for either 1 or 30 days (respectively, WD1 and WD30), prior to a test for cue-elicited METH-seeking. The description of the test for cue-elicited METH-seeking is provided in the main text.

**Tissue Dissection and Homogenization.** Immediately following the completion of the test for cue-elicited seeking, both METH and SHAM rats were rapidly decapitated, brains removed, sectioned using a rat brain mold (Braintree Scientific; Braintree MA, USA) and the prelimbic (PL) and infralimbic (IL) subregions of the medial prefrontal cortex (mPFC) were dissected out over ice. For this, angled incisions were made using thin forceps medial to the anterior corpus callosum, separating grey from white matter. The resulting triangular piece of grey matter tissue was then segmented as follows: the portion of the tissue that lay dorsal to the genu of the corpus callosum was considered anterior cingulate cortex (which was not examined herein), the portion of tissue running parallel with the genu of the corpus callosum labeled as the PL, and the tissue that ran parallel with the rostrum of the corpus callosum was considered IL and any remaining tissue ventral to the IL dissection was discarded (see Figure 2 for a depiction of the dissection sites). The same experimenter conducted all tissue dissection to ensure consistency both within and across experiments.

The extracted brain regions were homogenized in an ice-cold RIPA lysis buffer solution (50 mM Tris Base/Tris-HCl, pH 7.4; 150 mM NaCl; 0.5% Sodium Deoxycholate; 0.1% SDS) supplemented with Complete Protease Inhibitor Cocktail Tablets (Roche, Indianapolis, IN), 50 μL NaF (Sigma-Aldrich, St. Louis, MO), 50 μL 1 mM Sodium Orthovanadate (Sigma-Aldrich), and Phosphatase Inhibitor Cocktail 3 (Sigma-Aldrich). Following homogenization, the samples were placed on wet ice for at least 30 minutes to facilitate SDS-protein binding. The homogenized samples were then centrifuged at 10,500 RPM at 4°C for 20 minutes. Finally, the supernatant was stored upright in a -80°C freezer until immunoblotting.

**Immunoblotting.**

*Experiment 1: Biocorrelates of incubated methamphetamine-craving.* Incubated cocaine- and sucrose-craving are associated with increased indices of Akt/mTOR signaling, respectively, within the PL [2,3] and IL [4] and exhibit opposite or distinct changes in the expression of Group1 mGluRs and their Homer scaffolding proteins within these vmPFC subregions [4]. Thus, we first determined whether incubated METH-craving was associated with increased indices of Akt/mTOR activation within these mPFC subregions. For this, tissue from WD1-SHAM (METH-naive) rats (n=10) served as a baseline for protein expression and was compared to that of VEH-infused WD1-METH and WD30-METH animals from our behavioral study to examine for METH-induced changes in protein expression in early versus later withdrawal, respectively. The tissue from WD30-SHAM animals was also included in this experiment to control for any time-dependent changes in protein expression, unrelated to METH experience. Thus, four experimental groups were compared in Experiment 1: WD1-SHAM, WD1-METH, WD30-SHAM and WD30-METH. As in recent immunoblotting studies [5,6], the tissue from males and females were examined separately and the first 10 rats/treatment/sex were initially immunoblotted as this sample size was readily accommodated by our 12-lane electrophoresis gels (i.e., not all animals tested for behavior underwent immunoblotting). A total of 19 μL of homogenate was loaded into each well of 3-8% Tris-acetate gels (Invitrogen, Waltham, MA), separated via SDS-PAGE, and transferred onto PVDF membranes (Millipore, Billerica, MA). The membranes were blocked with 5% BSA/TBS (blocking buffer) for 1 hour at room temperature, and primary antibodies were prepared in the blocking buffer. The membranes were then incubated overnight at 4°C using the following mouse primary antibodies: mGluR1 (1:400 dilution; BD Biosciences; 610965), Homer1b/c (1:400 dilution; Santa Cruz Biotechnology; sc-25271), p(Ser473)-Akt1 (1:500 dilution; Cell Signaling Technology; 4051), mTOR (1:800 dilution; Cell Signaling Technology). Likewise, the following rabbit primary antibodies were employed: Homer2a/b (1:500 dilution; Synaptic Systems; 160- 203), Akt (1:500 dilution; Cell Signaling Technology; 9272), p(Ser2448)-mTOR (1:200 dilution; Cell Signaling Technology), mGluR5 (1:1000 dilution; Millipore; AB5675). Lastly, an anti-calnexin antibody (Rabbit, 1:1000 dilution, Enzo Life Sciences, ADI-SPA-860) was used as a loading control to ensure even protein loading.

Following the primary antibody incubation, unbound antibodies were removed by washing the membranes with Tris-Buffered Saline & Tween (TBST) three times for 5 minutes each, followed by rinsing with 1X TBS. The membranes were then incubated in a dark room for 1.5 hours with secondary antibodies, either goat anti-mouse IRDye 680RD (1:10,000 dilution, Li-Cor, 925-68070) or goat anti-rabbit IRDye 800CW (1:10,000 dilution, Li-Cor, 925-3221). After secondary incubation, the membranes were washed with TBST and rinsed with 1X TBS in a dark room before imaging using an Odyssey Infrared Imaging System (Li-Cor Biosciences, Lincoln, NE, USA). Statistical analysis of calnexin expression within the PL and the IL of the rats in this study indicated no significant group differences (Drug X WD ANOVAs: for male PL, all p>0.448; for female PL: all p>0.160; for male IL, all p>0.347; for female IL, all p>0.517). Thus, band intensities were quantified and then normalized to their corresponding calnexin signal. Normalized phosphoproteins were also compared to their respective normalized total protein levels. Note that blots exhibiting technical anomalies (e.g. lack of signal, misshapen band, high background, and bubbles from transfer) were excluded from the final statistical analyses of the data. As such, the sample sizes sometimes vary by 1-2 rats between brain regions for a particular protein or across proteins within a particular brain region.

*Experiment 2: Assessment of Akt/mTOR levels on Everolimus-treated METH-experienced rats.* To determine whether systemic Everolimus pretreatment reduced the activational state of Akt and/or mTOR, the PL and IL tissue from METH-experienced rats tested for cue-induced craving on WD30 (WD30-VEH, WD30-E1 and WD30-E10) were immunoblotted for mTOR, Akt, p(Ser2448)-mTOR and p(Ser473)-Akt in a second experiment, using identical immunoblotting procedures as described for Experiment 1. For this study, all rats from each of the 3 experimental groups were included. As such the sample sizes for the WD30-VEH groups are higher in Experiment 2 than in Experiment 1.

*Experiment 3: Assessment of Akt/mTOR levels on Everolimus-treated METH-naive rats.* To determine whether systemic Everolimus pretreatment reduced the activational state of Akt and/or mTOR in a METH-naive state, a distinct group of male METH-naive rats (n=8/pretreatment) were gavage-infused with either VEH or 10 mg/kg Everolimus and PL and IL tissue collected approximately 2.5 hours later (corresponding to the time that elapsed between gavage infusion and tissue collection in Experiment 2). As in Experiment 2, tissue was immunoblotted for mTOR, Akt, p(Ser2448)-mTOR and p(Ser473)-Akt, using identical immunoblotting procedures.

**Estrous Cycle Characterization during self-administration and cue testing.** Although estrous cycle is reported not to impact incubated METH-craving [7], incubated cocaine-craving is higher during estrus than other phases of the estrous cycle [6, 8-10]. Thus, we examined the influence of estrous phase on our behavioral measures. For the last five days of self-administration training and right after the cue test, epithelial cells around the vaginal canal were obtained using saline-presoaked cotton swab applicators (Fisher Scientific, Pittsburgh, PA). Cotton tip applicators were then rolled across Corning™ frosted microscope slides (Corning Incorporated, Corning, NY) and subsequently sprayed with fixative spray (Sigma-Aldrich). Slides then underwent Papanicolau staining and were subsequently cover-slipped (Fisher Scientific). Epithelial cell morphology was characterized under a microscope to correlate estrous cycle phase with METH intake and cue-elicited craving.

**Statistical Analyses.** The behavioral data for rats that self-administered METH were analyzed using a Sex X Group (WD1-VEH, WD30-VEH, WD30-E1 and WD30-E10) analysis of variance (ANOVA) with Tukey-HSD tests conducted for post-hoc analysis. The immunoblotting data were analyzed separately for each sex. To account for uneven protein loading and/or transfer, the signal for each protein of interest was normalized to its respective calnexin signal. To account for intermembrane variability, normalized signal for the respective control group (e.g., WD1-SHAM for study 1 and WD30-VEH for study 2) was then averaged within each membrane, and individual subject data were expressed as a percentage change relative to this average. For Study 1, statistical analysis of the immunoblotting data was performed using a two-way ANOVA with factors for Drug (SHAM and METH) X Withdrawal (WD1 and WD30) to assess differences in each protein of interest. In Study 2, immunoblotting data was analyzed using a one-way ANOVA to measure the effect of Everolimus pretreatment on protein levels. Unfortunately, the sample sizes for the females in different phases of the estrous cycle were either highly variable or insufficient (n=1-2) to sufficiently power a statistical analysis of the data from this aspect of the study. As such, the data were not analyzed by ANOVA, but are depicted in **Suppl. Figure 2** for visualization purposes. An extreme outlier was identified in the female WD30-VEH group for the number of active lever-presses on the test for incubated craving using the ± 3 × IQR rule as in previous work [3-6] and thus, the data for this variable was 90% winsorized [11]. All tests were two-tailed, with α set to 0.05.

**SUPPLEMENTAL RESULTS**

**Behavior of SHAM control rats.** The behavior of male and female SHAM control rats during both the 10-day operant-conditioning and the cue test phases of the study are summarized in **Suppl. Table 1**. No group differences were apparent with respect to the number of active lever-presses by SHAM controls during the 10-day operant-conditioning phase of the study (Sex X Withdrawal ANOVA: all p>0.104). However, by happenstance, the rats slated for tissue collection on WD30 emitted fewer inactive lever-presses than those slated for WD1 during the operant-conditioning phase of the study [Withdrawal effect: F(1,38)=7.798, p=0.008]. Further, during the cue test, rats slated for testing on WD30 happened to exhibit significantly higher levels of both active and inactive lever-pressing, compared to rats slated for testing on WD1 [for active lever, F(1,38)=5.407, p=0.026; for inactive lever, F(1,38)=6.421, p=0.016]. As behavior during the Cue Test was not directed at the cue-reinforced lever, we interpret this result as reflecting behavioral reactivity to being handled and returned to the operant chambers following a relatively lengthy period of confinement to the home cage.

**Methamphetamine intake and craving across estrous cycle.** As depicted in **Suppl. Figure 1**, there did not appear to be any overt relationship between estrous cycle phase and behavior expressed during operant-conditioning for METH reinforcement or during the Cue Test. As the total numbers of females within each estrous phase was variable during operant-conditioning (**Suppl. Figure 1A-C**), with samples sizes of 1-2 for certain estrous phases during Cue Testing (**Suppl. Figure 1D,E**), these data were not analyzed statistically.

**Correlates of methamphetamine-craving.** The results of correlational analyses between cue-elicited responding on the active lever during the cue test by METH-experienced rats and the expression of our various proteins of interest within the PL and the IL are summarized in **Suppl. Table 2**. As highlighted in the main text and illustrated in **Suppl. Figure 2**, METH-craving was correlated inversely with mGlu1 expression within the IL of both male and female rats, inversely with Homer2a/b expression within the female PL, positively with relative p(Ser473)-Akt expression within the female PL, and inversely with relative p(Ser2448)-mTOR expression within the male IL. No other significant correlations were detected.

**Supplemental References**

1. LaCrosse, A. L., O'Donovan, S. M., Sepulveda-Orengo, M. T., McCullumsmith, R. E., Reissner, K. J., Schwendt, M., & Knackstedt, L. A. (2017). Contrasting the Role of xCT and GLT-1 Upregulation in the Ability of Ceftriaxone to Attenuate the Cue-Induced Reinstatement of Cocaine Seeking and Normalize AMPA Receptor Subunit Expression. *The Journal of neuroscience*, *37*(24), 5809–5821. https://doi.org/10.1523/JNEUROSCI.3717-16.2017
2. Chiu, A. S., Kang, M. C., Huerta Sanchez, L. L., Fabella, A. M., Holder, K. N., Barger, B. D., Elias, K. N., Shin, C. B., Jimenez Chavez, C. L., Kippin, T. E., & Szumlinski, K. K. (2021a). Preclinical evidence to support repurposing everolimus for craving reduction during protracted drug withdrawal. *Neuropsychopharmacology*, *46*(12), 2090–2100. https://doi.org/10.1038/s41386-021-01064-9
3. Huerta Sanchez, L. L., Sankaran, M., Li, T. L., Doan, H., Chiu, A., Shulman, E., Shab, G., Kippin, T. E., & Szumlinski, K. K. (2023). Profiling prefrontal cortex protein expression in rats exhibiting an incubation of cocaine craving following short-access self-administration procedures. *Frontiers in Psychiatry*, *13*. https://doi.org/10.3389/fpsyt.2022.1031585
4. Cano, F. J., Denning, C. J. E., Udayashankar, H., Adler, S. D., Smith, K. E., Dang, V., Mohammadi, K., Reed Aparicio, A. T., Jotwani, A., Huerta Sanchez, L. L., & Szumlinski, K. K. (2025). Biomolecular correlates of incubated sucrose-seeking within ventromedial prefrontal cortex are sex- and subregion-selective. *Pharmacology Biochemistry and Behavior*, *256*, 174088. https://doi.org/10.1016/j.pbb.2025.174088
5. Huerta Sanchez, L. L., Tadros, M. G., Doan, H. H., Vo, S. V., Chaudhari, S. R., Li, T. L., James, P. B., Na, A. Y., Cano, F. J., Kippin, T. E., & Szumlinski, K. K. (2025). AMPA/kainate receptor activation within the prelimbic cortex is necessary for incubated cocaine-craving. *Frontiers in Psychiatry*, *16*. https://doi.org/10.3389/fpsyt.2025.1627477
6. Huerta Sanchez, L. L., Siao, N. M., Chaudhari, S. R., Barrios, J. E., Na, A. Y., Tadros, M. G., McConnell, M. L., Kaplan, R. M., Lane, C. R., Doan, H. H. T., Liger, A. B., Chou, T. C., Marcon, S., Cano, F. J., Kippin, T. E., & Szumlinski, K. K. (2026). Inactivation of NMDAR and CaMKII signaling within the prelimbic cortex blocks incubated cocaine- and sucrose-craving. *Neuropsychopharmacology*, 10.1038/s41386-025-02310-0. Advance online publication. https://doi.org/10.1038/s41386-025-02310-0
7. Funke, J. R., Hwang, E.-K., Wunsch, A. M., Baker, R., Engeln, K. A., Murray, C. H., Milovanovic, M., Caccamise, A. J., & Wolf, M. E. (2023a). Persistent neuroadaptations in the nucleus accumbens core accompany incubation of methamphetamine craving in male and female rats. *Eneuro*, *10*(3). https://doi.org/10.1523/eneuro.0480-22.2023
8. Kerstetter, K. A., Aguilar, V. R., Parrish, A. B., & Kippin, T. E. (2008a). Protracted time-dependent increases in cocaine-seeking behavior during cocaine withdrawal in female relative to male rats. *Psychopharmacology*, *198*(1), 63–75. https://doi.org/10.1007/s00213-008-1089-8
9. Nicolas, C., Russell, T. I., Pierce, A. F., Maldera, S., Holley, A., You, Z.-B., McCarthy, M. M., Shaham, Y., & Ikemoto, S. (2019a). Incubation of cocaine craving after intermittent-access self-administration: Sex differences and estrous cycle. *Biological Psychiatry*, *85*(11), 915–924. https://doi.org/10.1016/j.biopsych.2019.01.015
10. Corbett, C. M., Dunn, E., & Loweth, J. A. (2021a). Effects of sex and estrous cycle on the time course of incubation of cue-induced craving following extended-access cocaine self-administration. *Eneuro*, *8*(4). https://doi.org/10.1523/eneuro.0054-21.2021
11. Lee, B.K., Lessler, J., & Stuart, E. A. (2011). Weight Trimming and Propensity Score Weighting". *PLOS ONE,* 6(3), e18174. https://[doi](https://en.wikipedia.org/wiki/Doi_(identifier)):[10.1371/journal.pone.0018174](https://doi.org/10.1371%2Fjournal.pone.0018174).

**Supplemental Table 1:** Comparison of the average number of active and inactive lever-presses by male and female SHAM rats during the last 3 days of lever training and non-reinforced cue testing (Cue Test). The data represent the means ± SEMs of the number of animals indicated in parentheses.

|  |  | **Active Lever** | | **Inactive Lever** | |
| --- | --- | --- | --- | --- | --- |
| **Group** | **Sex** | **WD1** | **WD30** | **WD1** | **WD30** |
| **SHAM** | Male | 14.5 ± 3.1 (n=10) | 9.3 + 2.0 (n=10) | 7.4 + 1.1 (n=10) | 4.7 + 0.8 (n=10) |
|  | Female | 11.7 ± 2.7 (n=8) | 8.5 + 2.0 (n=11) | 10.4 + 1.7 (n=8) | 4.7 + 1.9 (n=11) |
| **SHAM**  **(Cue Test)** | Male | 4.6 ± 1.6 (n=10) | 13.3 ± 3.8 (n=10) | 0.6 + 0.2 (n=10) | 4.5 + 1.4 (n=10) |
|  | Female | 7.5 ± 2.2 (n=8) | 12.1 ± 3.0 (n=11) | 2.6 + 0.7 (n=8) | 6.6 + 2.4 (n=11) |

**Supplemental Table 2:** Correlations between cue-elicited METH-seeking and protein expression within the PL and the IL of male and female rats. Sample sizes are indicated in parentheses. Significant correlations indicated by an asterisk and bolded font. mGlu5M and mGlu5D=monomer and dimer form of mGlu5, respectively.

|  | **Female [n=19-20]** | | **Male [18-20]** | |
| --- | --- | --- | --- | --- |
| **Protein** | **PL** | **IL** | **PL** | **IL** |
| **mGlu1** | r=-0.145  p=0.542 | r=0.040  p=0.866 | r=-0.304  p=0.219 | **r=-0.536***  **p=0.015** |
| **mGlu5M** | r=-0.19  P=0.935 | r=-0.079  p=0.741 | r=-0.059  p=0.0816 | r=-0.278  p=0.249 |
| **mGlu5D** | r=0.027  p=0.913 | r=-0.204  p=0.389 | r=-0.025  p=0.921 | r=-0.203  p=0.403 |
| **Homer1b/c** | r=-0.083  p=0.729 | r=0.094  p=0.694 | r=-0.054  p=0.833 | r=-0.379  p=0.100 |
| **Homer2a/b** | r=-0.194  p=0.413 | r=0.068  p=0.774 | r=0.236  p=0.346 | r=-0.063  p=0.792 |
| **p-Akt** | r=0.222  p=0.347 | r=-0.006  p=0.979 | r=0.091  p=0.719 | r=-0.342  p=0.140 |
| **Akt** | r=-0.216  p=0.360 | r=0.217  p=0.358 | r=-0.035  p=0.889 | r=-0.142  p=0.551 |
| **p-Akt/Akt** | **r=0.460***  **p=0.041** | r=-0.294  p=0.207 | r=-0.087  p=0.733 | r=-0.269  p=0.252 |
| **p-mTOR** | r=-0.007  p=0.976 | r=0.120  p=0.613 | r=-0.134  p=0.584 | r=-0.170  p=0.474 |
| **mTOR** | r=-0.186  p=0.431 | r=0.158  p=0.506 | r=0.090  p=0.714 | r=0.089  p=0.709 |
| **p-mTOR/mTOR** | r=0.062  p=0.795 | r=0.062  p=0.795 | r=0.093  p=0.715 | **r=-0.511***  **p=0.021** |

**Supplemental Figure Legends**

**Supplemental Fig. 1:** Characterizing estrous phase following METH intake and craving. Over the last 5 days of the 6-hr METH self-administration period, the estrous stage was identified for each rat to determine potential influences in **(A)** active and **(B)** inactive lever presses, as well as **(C)** reinforcers earned. The estrous phase of each rat was also identified following the 2-hr cue test. No group differences are detected

**Supplemental Fig. 2:** Sex differences in the protein correlates of METH-seeking. In the PL, cue-elicited METH-seeking was **(A)** positively correlated with p(Ser473)-Akt:Akt in females. In the IL, cue-elicited METH-seeking was negatively correlated with **(B)** p(Ser2448)-mTOR:mTOR and with **(C)** mGlu1 levels in males. Sample sizes are indicated in parentheses, and the results from the correlational analyses are included in each panel.

**Supplemental Fig. 3:** Immunoblotting results for the effects of Everolimus pretreatment on mTOR and Akt expression within the PL and IL of METH-naive male and female rats. PL (Female, Male, respectively): **(A,D)** p(Ser2448)-mTOR:mTOR, **(B,E)** p(Ser2448)-mTOR, **(C,F)** total mTOR, **(G,J)** p(Ser473)-Akt:Akt, **(H,K)** p(Ser473)-Akt, **(I,L)** total Akt. IL (Female, Male, respectively): **(M,P)** p(Ser2448)-mTOR:mTOR, **(N,Q)** p(Ser2448)-mTOR, **(O,R)** total mTOR, **(S,V)** p(Ser473)-Akt:Akt, **(T,W)** p(Ser473)-Akt, **(U,X)** total Akt. The data are expressed relative to WD30-VEH and represent the means ± SEMs of the individual rats indicated (n=5-12). A cartoon depicting the tissue PL and IL dissection is provided in Figures 3-5 of the main text and representative immunoblots from this experiment are provided. No group differences were detected.
